# Mysterious girls: The biology of female *Cryptosporidium* parasites

**DOI:** 10.1371/journal.ppat.1014678

**Published:** 2026-10-12

**Authors:** Sarah Stevens, Peyton Goddard, Katelyn A. Walzer, Mattie Christine Pawlowic

**Affiliations:** 1 Faculty of Life Sciences, University of Dundee, Dundee, United Kingdom; 2 Department of Biological Sciences, Eukaryotic Pathogens Innovation Center (EPIC), Clemson University, Clemson, SC, United States of America; Institute of Parasitology, Biology Centre, Czech Academy of Sciences, CZECHIA

## *Cryptosporidium* biology and current challenges

*Cryptosporidium* spp. is a leading cause of diarrhoeal disease, for which there are no effective drugs or vaccines. The parasite infects a diverse range of vertebrate hosts, with human infection causing significant global morbidity and mortality. In young, malnourished children and the immunocompromised, disease is severe and often fatal [1]. Infection in these patients can also be chronic and cause wasting or growth stunting. Therefore, there is an urgent need for new interventions. New tools to study the parasite in the laboratory have advanced our understanding of the molecular mechanisms that underpin the parasite life cycle and provide more insight into the biology of this parasite with global health impact. Most of our understanding of the parasite’s biology comes from studies conducted with *C. parvum*, as it is the most tractable species to study in the laboratory. We have focussed predominantly on knowledge gained from this species. Here we describe recent developments in our understanding of the parasite life cycle, focussing on sexual stages, and highlight some of the remaining gaps in our understanding.

*Cryptosporidium* completes its entire life cycle in a single host. Infection begins when a host ingests the transmissible form of the parasite, called the oocyst. The oocyst is made of an environmentally resilient shell or “wall” that protects four internal sporozoites. Once inside the gastrointestinal tract, the oocyst “hatches”, or excysts, releasing the sporozoites. The sporozoites invade and infect the epithelial cells that line the gut. Cryptosporidia are obligate intracellular parasites and replicate inside a parasitophorous vacuole. To do this, they attach to the surface of a gut cell and “pull” the membrane of the cell around themselves to form the parasitophorous vacuole [2]. Inside the vacuole, the sporozoite develops into a meront, replicating the nucleus and organelles. After about 12 hours, they finish replicating and then divide to produce eight motile, merozoite forms. These merozoites egress from the parasitophorous vacuole and from the host cell and invade a nearby gut epithelial cell. Each merozoite repeats this asexual replication cycle two more times, each time producing eight more merozoites. *C. parvum* merozoites produced from the third round of asexual replication are committed to sexual stages without a morphologically distinct 4N type II meront [3]. Whilst four-merozoite meronts have been described in *C. muris* [4] and *C. mortiferum* [5], data from live-cell imaging approach (*in vitro* infection of HCT-8 cells) used by English *et al.,* and the single-cell RNASeq data from *in vitro* and *in vivo* infections by Walzer *et al.* [6] suggest direct development of type I meronts into the sexual stage. Similar experimentation in other *Cryptosporidium* species is required to confirm the absence of type II meronts.

The mechanisms for why and how the parasites commit to sexual stages after exactly three rounds of asexual replication are still unknown. Following asexual replication, the sexually committed merozoite then invades a new epithelial cell and forms either a macrogamont, which develops a single female gamete, or a microgamont, which develops 16 male gametes. Once the males have matured, they egress and seek out a female parasite. Female parasites do not egress but remain intracellular and mature here until they are fertilised. Fertilisation produces a parasite zygote that goes through meiosis (DNA replication and segregation), construction of the oocyst wall, and sporogony (replication and segregation of organelles) to produce four haploid sporozoites, contained within the oocyst wall (Fig 1). Co-culture of *C. parvum* with HCT-8 cells does not allow for the observation of fertilisation and onwards zygote and oocyst development. This renders parts of the parasite’s sexual life cycle (sexual commitment, sexual development, fertilisation, meiosis, sporogony, and oocyst development) particularly ‘mysterious’. A phenomenal burden falls upon the female parasite to prepare for oocyst construction prior to fertilisation. Much of female development, and the mechanism of oocyst wall-building, remains to-date uncharacterised.

**Fig 1:**
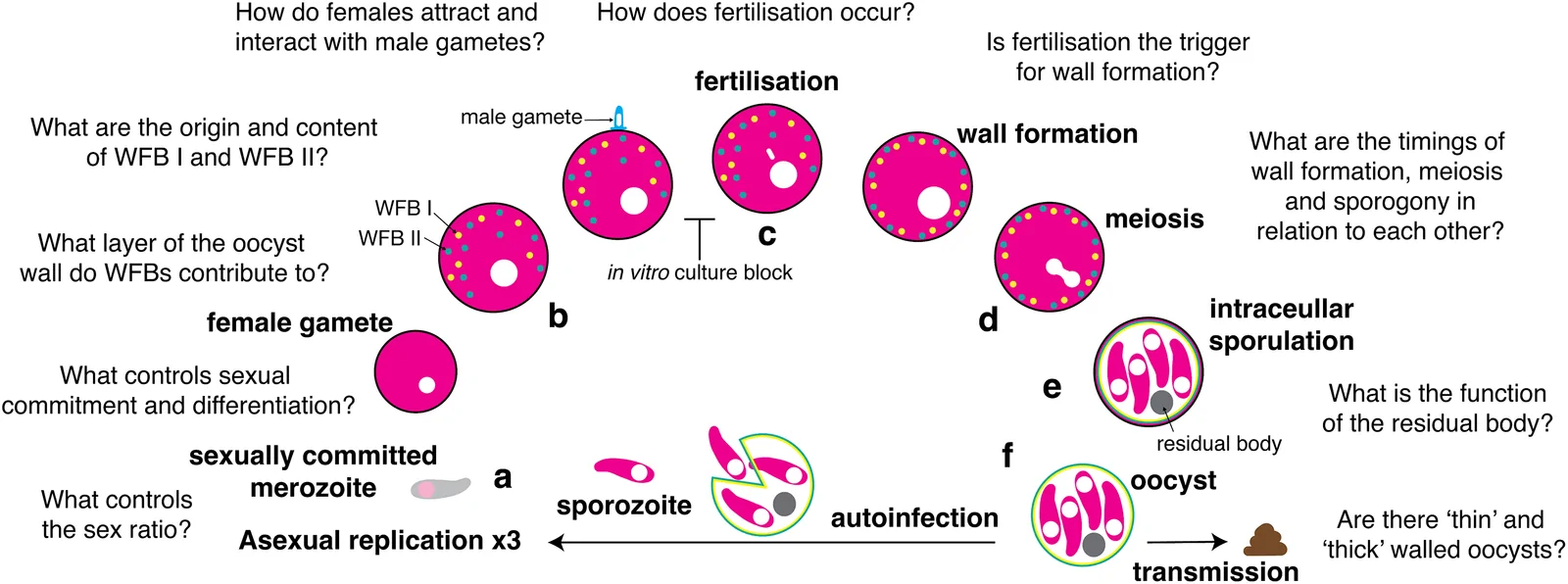
Unanswered questions about *Cryptosporidium* female sexual development. Merozoites produced from the third round of asexual replication are committed to sexual stages **(a)** pink nucleus of the grey merozoites indicates female fate. This merozoite invades a gut cell and starts to mature. For simplicity, the cytoplasm of the female gamete is illustrated in pink, its nucleus in white, and the parasite plasma membrane in black. The female gamete synthesises and then stores components of the oocyst wall inside Wall Forming Body organelles (WFB). There are two types of WFBs and it is hypothesised that these correspond to the two layers of the oocyst wall (yellow and teal) **(b)**. Multiple WFBs of each type are prepared prior to fertilisation. Upon fertilisation by a male gamete (blue) **(c)**, wall formation, meiosis and intracellular sporulation occur. The resulting oocyst forms inside the gut cell **(d, e)**. The exact sequence of these events and how they are regulated is unknown. Unlike other coccidia, *Cryptosporidium* oocysts do not require further maturation outside the body and are immediately infectious and ready to reinitiate the life cycle. Oocysts are released from the host cell and either shed in the faeces (transmission) or excyst in the gut, release their sporozoites, and reinfect the current host (autoinfection) **(f)**.

## Sexual commitment: To be, or not to be?

For the third generation of merozoites, the commitment to sexual development is mandatory [3,6]. Live cell imaging of *C. parvum*-infected HCT-8 cells desmonstrated that merozoites from a single meront give rise to both male and female parasites [3]. Out of the 8 merozoites in a meront, approximately 2–3 will be male and the remaining 5–6 will be female [3]. This generates an excess ratio of small and motile microgametes to find and fertilise the female [7]. Sexual commitment is governed by an intrinsic and non-chromosomal mechanism. One of the factors known to be involved is the transcription factor Myb-M [6]. This transcription factor is expressed early during male development. Experimental “overexpression” of this transcription factor in *C. parvum* is enough to initiate differentiation into males. An equivalent trigger for female commitment has not yet been identified, suggesting that female fate may be the default programming for sexual differentiation.

## A femininomenon

Female parasites have a unique and complex developmental progression. Following commitment, macrogametes undergo significant preparations for fertilisation, meiosis, oocyst wall construction, and sporulation. To accomplish all these feats, females have a large nucleus, reflecting their status as the most transcriptionally and translationally active life cycle stage [3]. Early on in development, females express putative transporters, membrane proteins, and secreted proteins that are hypothesised to be involved in fertilisation. These may be expressed early to facilitate male attachment, entry, and fertilisation. They later have two waves of expression of genes involved in the construction of the oocyst wall.

The oocyst wall is comprised of a glycocalyx, an outer wall made of acid-fast lipids, and an inner wall made of glycoproteins [8]. These components are synthesised and stored in female-exclusive organelles called Wall Forming Bodies (WFBs). There are two subtypes of these organelles, which are differentiated by electron density [9]. It is hypothesised that WFB Type I and Type II are secreted sequentially, with the contents of WFB Type I secreted first and used to build the outer layer of the oocyst wall, and WFB Type II the inner layer of the oocyst wall. Currently, *COWP1* is the only confirmed marker for WFB Type I [10]. *COWPs* 6 and 8 have a punctate localisation in developing females, suggesting they also localise to WFBs [11].

*C. parvum* female transcriptomics and oocyst proteomics data have generated a comprehensive list of the candidate WFB proteins [6,7,12], but more work is needed to understand how these organelles are synthesised, what they contain, and how the secretion of their contents is regulated. From the C*. parvum* single-cell atlas, we know that the first wave of gene expression contains the family of *Cryptosporidium* Oocyst Wall Proteins (*COWP*s) [11] and accessory enzymes (proteases and protein modifiers) for building the proteinaceous shell. The second wave expresses glycosyltransferases and other secreted proteins (mucins and C-type lectin) [6]. These components are secreted in the space between the parasite plasma membrane and the host cell membrane to build the oocyst wall while the parasite is still inside the gut cell [9]. Having oocyst wall components ready ‘just in time’ allows these proteins to be secreted from WFBs and used to construct the oocyst wall shortly after fertilisation, presumably to keep up with the fast turnover of the epithelial host cells.

Females also synthesise proteins that are required by the progeny sporozoites. Genetic crosses involving isolates tagged with different fluorescent proteins have shown that maternal cytoplasmic proteins persist in the sporozoites of the F1 progeny [13–15]. These include proteins involved in assembly of dense granule organelles, which function in remodelling of the host cell after the rhoptry has been discharged during invasion [16,17]. Females also express the proteins of the crystalloid body, the function of which is still unknown, but likely involved in sporogony [18].

Female RNA-binding proteins may act as post-transcriptional modulators or repressors. It may be that the decay and accumulation of a particular protein, RNA, or metabolite is required for development. For example, *F-RBP* (cgd4_130) is a maternal RNA-binding protein expressed in early females and was recently shown to bind to female RNA transcripts, with conditional knockouts *in vivo* producing ‘sterile’ sporozoites [19]. *F-RBP* may be essential for regulation of maternally inherited RNA needed for successful development of infectious sporozoites in the next generation [19].

### What controls female development?

Female development appears tightly regulated, yet few proteins have been elucidated as key players in this process. Two Apetala-2-type (ApiAP2) DNA-binding proteins are female-specific and required for female differentiation (ApiAP2-1; cgd2_3490) and oocyst shedding (*ApiAP2-F*; cgd4_1110) [20,21]. Insulinase-like protease 1 (*INS1*; cgd1_1680) is also involved in female development and oocyst shedding [22]. These genes collectively suggest a level of transcriptional control in female development.

Females also show reduced expression of ribosomal proteins, indicative of translational repression [20]. Drawing from known apicomplexan mechanisms, we hypothesise transcriptional modifications are also involved in female development. This might include epigenetic switches through chromatin remodelling, which seems likely given the expansion of the nucleus. Whatever combination of molecular mechanisms govern female development, they are fundamental to complete the parasite life cycle and set up the next generation, so deserve attention as potential targets for intervention.

## Parasite sex… and what happens next?

*In vitro* culture of *C. parvum* with adenocarcinoma cell lines (HCT-8 cells) allows for the observation of the asexual life cycle stages and gametocytogenesis but has left the processes of fertilisation, meiosis, sporulation, and oocyst formation understudied. How females attract and interact with the male gametes during fertilisation is therefore a mystery (Fig 1c).

Fertilisation is the presumed trigger that initiates oocyst wall formation, sporogony and meiosis, but without a culture system that enables observation of this process, we lack evidence of this. Additionally, the signals that regulate post-fertilisation zygote development are unknown across coccidia. In *Cryptosporidium,* it appears that meiotic divisions precede and partially overlap with the deposition of the oocyst wall [7,9]. Glideosome components, microneme and rhoptry proteins are not expressed by *in vitro* females, but are *in vivo*, suggesting they are only synthesised after fertilisation [6]. *In vivo*, females have everything prepared for the zygote to immediately undergo meiosis and sporogony to form infectious oocysts.

### Meiosis

To form four haploid sporozoites, the zygote nucleus undergoes meiosis prior to sporulation (Fig 1d). Genes related to meiosis and DNA repair are expressed by female parasites [6,7], including DNA Meiotic Recombinase (*DMC1*; cgd7_1690) which is expressed in the nuclei of female parasites [23]. Under experimental conditions, *C. parvum* chromosomes exhibit Mendelian inheritance patterns, with a high frequency of meiotic recombination at the loci examined [24]. Frequent sex and high recombination between genetically distinct parasites can potentially contribute to the evolution and spread of traits like virulence or drug resistance, for example, clofazimine resistance observed between *C. parvum* subtypes [25].

### Sporogony

In contrast to other apicomplexans, *Cryptosporidium* oocysts are shed from the host epithelium fully sporulated (Fig 1e). Sporogony begins with the formation of the residual body separating from the sporont [9]. The crystalloid body, a multi-vesicular structure localised to the basal end of sporozoites, is essential for transmission in *Plasmodium* [18], and has been implicated in oocyst formation and shedding [21]. Putative components of this organelle have been identified, and a few have been validated, but their function is still unknown [12]. Division of the nuclei, membrane and organelles during cytokinesis has not yet been observed in detail but presumably responds to different triggers from other Coccidia (i.e., not environmental). The result of sporogony is four haploid sporozoites housed within a protective shell - the oocyst.

## What makes a shell?

Oocyst biogenesis is exclusive to female parasites. They must synthesise and store all the components to produce the oocyst wall; a multilayered protective shell. Underneath an external glycocalyx is the outer bilayer of acid-fast lipids. The outer wall is composed of acid-fast lipids that are hypothesised to protect against chemical disinfection, including bleach [26]. An inner protein layer of cysteine-rich glycoproteins is proposed to form an extensive disulphide-bonded protein scaffolding network, responsible for the structural strength [27]. This bi-layered shell is conserved across Coccidia, with *Cryptosporidium* exhibiting the simplest oocyst model given it lacks internal sporocyst structures.

### Thick or thin?

Because oocysts sporulate in the gut, they can either be excreted and transmitted to another host or can excyst and reinfect the current host. This reinfection facilitates the chronic, deadly disease experienced by immunocompromised patients and malnourished children (Fig 1f). A historically proposed model for Cryptosporidia is that there are two different types of oocysts: ‘thin-walled’ oocysts that excyst within the current host and drive autoinfection, and ‘thick-walled’ oocysts that are excreted and transmitted [9]. A few electron micrograms serve as the evidence to support the existence of thin-walled oocysts. However, there is no suggestion that females have different transcriptional profiles that lead them to produce thin-walled oocysts [6]. We hypothesise that all oocysts are ‘thick-walled’ and capable of surviving in the environment, but that a small number of these oocysts excyst inside the gut before excretion. Due to the exponential nature of the asexual life cycle, even a small number of reinfections can result in high levels of infection.

## Girl power

*Cryptosporidium* females undergo a remarkable transformation during their sexual development that enables them to set up the next generation of parasites. Their unique biology is largely unexplored yet is crucial for both current infection and onward transmission. Improved *in vitro* culture methods, such as organoid co-culture [13], have made post-fertilisation investigation possible. *Cryptosporidium* females can give us insight into the shared female apicomplexan mechanisms for oocyst biogenesis. Understanding the process of oocyst biogenesis, driven by female powerhouse gametes, can identify factors essential for transmission and perhaps offer appealing targets for new interventions. It is time to stop overlooking and underestimating *Cryptosporidium* females - a true femininomenon of parasite biology.

## References

[1] Kotloff KL, Nataro JP, Blackwelder WC, Nasrin D, Farag TH, Panchalingam S, et al. Burden and aetiology of diarrhoeal disease in infants and young children in developing countries (the Global Enteric Multicenter Study, GEMS): A prospective, case-control study. Lancet. 2013;382(9888):209–22. doi: 10.1016/S0140-6736(13)60844-2 23680352

[2] Guérin A, Roy NH, Kugler EM, Berry L, Burkhardt JK, Shin J-B, et al. *Cryptosporidium* rhoptry effector protein ROP1 injected during invasion targets the host cytoskeletal modulator LMO7. Cell Host Microbe. 2021;29(9):1407-1420.e5. doi: 10.1016/j.chom.2021.07.002 34348092 PMC8475647

[3] English ED, Guérin A, Tandel J, Striepen B. Live imaging of the *Cryptosporidium parvum* life cycle reveals direct development of male and female gametes from type I meronts. PLoS Biol. 2022;20(4):e3001604. doi: 10.1371/journal.pbio.3001604 35436284 PMC9015140

[4] Melicherová J, Ilgová J, Kváč M, Sak B, Koudela B, Valigurová A. Life cycle of *Cryptosporidium muris* in two rodents with different responses to parasitization. Parasitology. 2014;141(2):287–303. doi: 10.1017/S0031182013001637 24128742

[5] Tůmová L, Ježková J, Prediger J, Holubová N, Sak B, Konečný R, et al. *Cryptosporidium mortiferum* n. sp. (Apicomplexa: Cryptosporidiidae), the species causing lethal cryptosporidiosis in Eurasian red squirrels (*Sciurus vulgaris*). Parasit Vectors. 2023;16(1):235. doi: 10.1186/s13071-023-05844-8 37454101 PMC10349434

[6] Walzer KA, Tandel J, Byerly JH, Daniels AM, Gullicksrud JA, Whelan EC, et al. Transcriptional control of the *Cryptosporidium* life cycle. Nature. 2024;630(8015):174–80. doi: 10.1038/s41586-024-07466-1 38811723 PMC12057246

[7] Tandel J, English ED, Sateriale A, Gullicksrud JA, Beiting DP, Sullivan MC, et al. Life cycle progression and sexual development of the apicomplexan parasite *Cryptosporidium parvum*. Nat Microbiol. 2019;4(12):2226–36. doi: 10.1038/s41564-019-0539-x 31477896 PMC6877471

[8] Samuelson J, Bushkin GG, Chatterjee A, Robbins PW. Strategies to discover the structural components of cyst and oocyst walls. Eukaryot Cell. 2013;12(12):1578–87. doi: 10.1128/EC.00213-13 24096907 PMC3889564

[9] Current WL, Reese NC. A comparison of endogenous development of three isolates of *Cryptosporidium* in suckling mice. J Protozool. 1986;33(1):98–108. doi: 10.1111/j.1550-7408.1986.tb05567.x 3959014

[10] Spano F, Puri C, Ranucci L, Putignani L, Crisanti A. Cloning of the entire COWP gene of *Cryptosporidium parvum* and ultrastructural localization of the protein during sexual parasite development. Parasitology. 1997;114 (Pt 5):427–37. doi: 10.1017/s0031182096008761 9149414

[11] Bacchetti R, Stevens S, Lemgruber L, Gonzalez Oliva MA, Sands EM, Alexandrou K, et al. *Cryptosporidium* oocyst wall proteins are true components of the oocyst wall and COWP8 is not required for parasite transmission. PLoS Pathog. 2025;21(10):e1013561. doi: 10.1371/journal.ppat.1013561 41129551 PMC12548878

[12] Guérin A, Strelau KM, Barylyuk K, Wallbank BA, Berry L, Crook OM, et al. *Cryptosporidium* uses multiple distinct secretory organelles to interact with and modify its host cell. Cell Host Microbe. 2023;31(4):650-664.e6. doi: 10.1016/j.chom.2023.03.001 36958336

[13] Wilke G, Funkhouser-Jones LJ, Wang Y, et al. (2019) A stem-cell-derived platform enables complete *Cryptosporidium* development in vitro and genetic tractability. Cell Host Microbe 26: 123–34.e8. doi: 10.1016/j.chom.2019.05.007 PMC6617391 31231046

[14] Hanna JC, Corpas-Lopez V, Seizova S, Colon BL, Bacchetti R, Hall GMJ, et al. Mode of action studies confirm on-target engagement of lysyl-tRNA synthetase inhibitor and lead to new selection marker for *Cryptosporidium*. Front Cell Infect Microbiol. 2023;13:1236814. doi: 10.3389/fcimb.2023.1236814 37600947 PMC10436570

[15] Shaw S, Cohn IS, Baptista RP, Xia G, Melillo B, Agyabeng-Dadzie F, et al. Genetic crosses within and between species of *Cryptosporidium*. Proc Natl Acad Sci U S A. 2024;121(1):e2313210120. doi: 10.1073/pnas.2313210120 38147547 PMC10769859

[16] Puthenpurackal A, Moreno Sanchez S, Schaer T, Falconnet L, Niu C, Vadas O, et al. *Cryptosporidium* secreted proteins form a complex layered interface with the host cell. PLoS Pathog. 2026;22(5):e1014212. doi: 10.1371/journal.ppat.1014212 42133745 PMC13241007

[17] Cohen A, Bergner T, Guérin A. *Cryptosporidium* sequentially remodels its single rhoptry into the host interface. bioRxiv. 2026. doi: 10.64898/2026.07.21.739819

[18] Dessens JT, Tremp AZ, Saeed S. Crystalloids: Fascinating Parasite Organelles Essential For Malaria Transmission. Trends Parasitol. 2021;37(7):581–4. doi: 10.1016/j.pt.2021.04.002 33941493

[19] Daniels AM, Shaw S, Nötzel C. Ablation of a maternal Cryptosporidium mRNA-binding protein results in sterile sporozoites. bioRxiv. 2026. doi: 10.64898/2026.09.01.748511

[20] Hasan MM, Mattice EB, Teixeira JE, Jumani RS, Stebbins EE, Klopfer CE, et al. Cryptosporidium life cycle small molecule probing implicates translational repression and an Apetala 2 transcription factor in macrogamont differentiation. PLoS Pathog. 2024;20(4):e1011906. doi: 10.1371/journal.ppat.1011906 38669269 PMC11078545

[21] Tandel J, Walzer KA, Byerly JH, Pinkston B, Beiting DP, Striepen B. Genetic ablation of a female-specific apetala 2 transcription factor blocks oocyst shedding in *Cryptosporidium parvum*. mBio. 2023;14(2):e0326122. doi: 10.1128/mbio.03261-22 36786597 PMC10233709

[22] Xu R, Feng Y, Xiao L, Sibley LD. Insulinase-like protease 1 contributes to macrogamont formation in *Cryptosporidium parvum*. mBio. 2021;12(2):e03405-20. doi: 10.1128/mBio.03405-20 33688009 PMC8092296

[23] Jumani RS, Hasan MM, Stebbins EE, Donnelly L, Miller P, Klopfer C, et al. A suite of phenotypic assays to ensure pipeline diversity when prioritizing drug-like *Cryptosporidium* growth inhibitors. Nat Commun. 2019;10(1):1862. doi: 10.1038/s41467-019-09880-w 31015448 PMC6478823

[24] Kimball A, Funkhouser-Jones L, Huang W, Xu R, Witola WH, Sibley LD. Mendelian segregation and high recombination rates facilitate genetic analyses in *Cryptosporidium parvum*. PLoS Genet. 2024;20(6):e1011162. doi: 10.1371/journal.pgen.1011162 38885280 PMC11213348

[25] Buenconsejo GY, Shaw S, Xiao R, Balestra AC, O’Dea KM, Jiang P, et al. Genomic heterogeneity of NAD(P)H dehydrogenase predisposes *Cryptosporidium* to clofazimine resistance. Nat Microbiol. 2026;11(6):1559–72. doi: 10.1038/s41564-026-02331-5 42129495 PMC13236581

[26] Bushkin GG, Motari E, Carpentieri A. Evidence for a structural role for acid-fast lipids in oocyst walls of *Cryptosporidium*, *Toxoplasma*, and *Eimeria*. mBio. 2013;4:e00387-13. 10.1128/mBio.00387-13 PMC3760245 24003177

[27] Jenkins MB, Eaglesham BS, Anthony LC, Kachlany SC, Bowman DD, Ghiorse WC. Significance of wall structure, macromolecular composition, and surface polymers to the survival and transport of *Cryptosporidium parvum* oocysts. Appl Environ Microbiol. 2010;76(6):1926–34. doi: 10.1128/AEM.02295-09 20097810 PMC2838015

